# Efficacy and safety of emulsified microsomal ferric pyrophosphate vs. Ferrous Ascorbate in pregnancy with iron-deficiency anemia- a randomized, comparative study

**DOI:** 10.1038/s41598-025-23353-9

**Published:** 2025-11-12

**Authors:** Ameet Patki, G. S. Jyothi, Vidya Thobbi, Ankita Srivastav, Gayatri Ganu, Alok S. Shah

**Affiliations:** 1 Fertility Associates Clinic, 81, 4th Floor, Gupte House, Police Station, Swami Vivekananda Rd, near Khar, Ram Krishna Nagar, Khar West, Mumbai, 400054 Maharashtra India; 2 Department of Obstetrics and Gynaecology, Ramaiah Medical College and Hospitals, Bengaluru, 560054 India; 3 Department of Obstetrics and Gynaecology, Al-Ameen Medical College, Bijapur, Vijayapura, 586108 Karnataka India; 4 Generex Pharmassist Pvt. Ltd., 4th floor, DGP House, 88C, Old Prabhadevi Road, Prabhadevi, Mumbai, 400025 India; 5 Mprex Healthcare Pvt. Ltd., Office Number 501, 514 Crossroads, Bhumkar Square, Wakad, Pune, India

**Keywords:** Iron deficiency anemia, Hemoglobin, M cells, Ferrous ascorbate, Emulsified ferric pyrophosphate, Pregnancy, Diseases, Gastroenterology, Health care, Medical research

## Abstract

This study evaluates the efficacy and safety of emulsified microsomal Ferric pyrophosphate (EMFP/SunActive™ Fe, 27 mg elemental iron) versus Ferrous Ascorbate (100 mg elemental iron) in second-trimester pregnant women with iron-deficiency anemia (IDA) for 4 weeks. Pregnant women aged 20–35 years with a singleton pregnancy, hemoglobin (Hb) 9–10.5 g/dL, and ferritin < 15 mcg/L were enrolled. The test group showed zero adverse effects vs. the control group, having 11.1% adverse events. The gastrointestinal(GI) adverse symptoms, including nausea, dark stools, and hyperacidity, were reported only in the Ferrous Ascorbate group, indicating superior tolerability and safety of EMFP tablets. Both groups showed similar improvements in Hb (Δ2.63 g/dL vs. Δ2.62 g/dL) and serum ferritin (61.09% vs. 61.92%). Reticulocyte hemoglobin (RET-He) increased by 20.5% in the test group and 16.2% in the control group, with no significant difference. Clinical symptoms such as dizziness, fatigue, and palpitations improved with greater magnitude in the test group. It was inferred that the test group receiving EMFP was as effective as the control group in improving efficacy endpoints at a significantly lower dose (1/3rd dose compared to ascorbate). EMFP showed better tolerability, safety and compliance, making it a promising option for managing IDA in pregnant women.

## Introduction

Anemia is characterized by a reduction in hemoglobin (Hb) and red blood cell (RBC) concentration, leading to decreased oxygen-carrying capacity. Its occurrence is influenced by factors such as age, sex, altitude, smoking habits, and pregnancy^1,2^. When Hb levels drop below 11 g/dL, it is considered anemia during pregnancy. Further classification of Hb levels is as follows: mild (10–10.9 g/dL), moderate (7–9.9 g/dL), and severe (less than 7.0 g/dL)^3^. Pregnant women and young children are particularly vulnerable to anemia, a major public health problem worldwide. Nearly 40% of pregnant women experienced anemia in 2016, as reported by the World Health Organization (WHO)^2^. In Southeast Asia, anemia is a leading cause of maternal mortality. Approximately 80% of the maternal fatalities in South Asia occur in India. According to the 2019–2021 National Family Health Survey-5 (NFHS-5), approximately 57% of women of reproductive age were found to be anemic^4^. Anemia occurs during pregnancy in 45.7% of pregnant women in urban areas and 52.1% of pregnant women in rural regions in India, according to cross-sectional research^5^.

Globally, iron deficiency remains the leading cause of anemia in pregnant women. During pregnancy, maternal iron demand increases significantly, with daily requirements rising from 0.8 mg in the first trimester to 7.5 mg in the third trimester. This heightened demand is driven by the need to support fetal development, expand maternal red blood cell mass, and compensate for peripartum iron losses. Consequently, anemia is highly prevalent during pregnancy due to the substantial physiological adaptation required to meet these increased iron demands^6,7^. This issue is exacerbated by factors like early childbearing, low intervals between pregnancies, high rates of multiple pregnancies, and limited access to prenatal care and supplements^8^.

Unrecognised and untreated iron-deficiency anemia (IDA) can have severe consequences for both maternal and fetal health. Chronic iron deficiency impairs overall well-being, leading to fatigue, reduced functional capacity, and a range of clinical symptoms, including pallor, breathlessness, palpitations, headaches, dizziness, and irritability. Emerging evidence highlights a strong association between maternal anemia and an increased risk of intrauterine growth restriction (IUGR), preterm birth, low birth weight (LBW), pre-eclampsia, postpartum hemorrhage, maternal sepsis, and mortality of both mother and child^7,9^. These risks underscore the critical importance of early detection and management of IDA during pregnancy.

IDA treatment options include both oral and intravenous (IV) iron replacement. Oral iron therapy, despite being convenient, inexpensive, and effective for stable patients, often presents challenges due to gastrointestinal (GI) intolerance. Common iron salt supplements like sulfate, gluconate, fumarate, ascorbate, or carbonyl iron are prescribed, yet long-term oral iron treatment is hindered by taste alterations and GI symptoms such as nausea, vomiting, constipation, metallic taste, and abdominal discomfort. These issues frequently necessitate dose adjustments, prescription changes, non-adherence, or treatment discontinuation. Moreover, iron protein succinylate and iron polymaltose complex, while available, are infrequently utilised due to poor absorption, high cost, and increased dosage requirements. Meta-analyses comparing oral and IV iron supplementation indicate a notable incidence of GI adverse events, affecting around 32% of participants receiving oral iron^9–11^. In contrast, although IV iron offers more reliable and rapid distribution, it does not necessarily lead to a quicker increase in Hb levels and is associated with its adverse effects, including nausea, anaphylaxis, and the risk of extravasation^12^.

For the well-being of the mother as well as the baby, it is crucial to take an iron supplement during pregnancy to prevent anemia, which is a major problem worldwide. Iron supplementation is an important therapeutic strategy because recent research has demonstrated that it can greatly enhance hematological parameters^13^. But the efficacy seen with the use of conventional iron is variable due to associated side effects like GI intolerance and, in turn, a lack of compliance.

The popular iron salt in the Indian subcontinent is Ferrous Ascorbate. However, its use has been associated with increased GI issues. Next-generation encapsulated irons represent a potential solution to this problem. However, no direct clinical trials comparing Ferrous Ascorbate and next-generation encapsulated iron have been reported.

Conventional iron is absorbed through DMT-1 channels, which can be rate-limiting and lead to free iron. This free iron is a primary cause of the side effects associated with conventional iron supplementation, as it can contact the intestinal mucosa and cause significant GI discomfort.

In contrast, SunActive™ Fe features micronized particles (0.3–0.5 μm) that are optimally absorbed via microfold cells (M cells), bypassing the conventional absorption route^14^. This allows SunActive™ Fe to enter the systemic circulation through the lymphatic system, preventing free iron from interacting with the intestinal mucosa and thereby significantly reducing gastrointestinal discomfort, enabling more consistent absorption and bioavailability, with potentially faster hemoglobin rise^15^.

Furthermore, its stable physical properties eliminate precipitation, iron taste, and colour changes while maintaining stability against heat, pH, and oxidation^16^. It is the only micronised form of Ferric pyrophosphate recommended by the WHO and UN^17^.

Hence, it is critical to compare the two to determine the most appropriate form and dose of iron to successfully replenish iron stores, maintain normal Hb levels with minimal side effects, and ensure good patient compliance.

The present study was designed to evaluate the efficacy and safety of SunActive™ Fe (27 mg) compared to Ferrous Ascorbate tablets (100 mg) in second-trimester pregnant women with IDA. This approach aligns with the need for optimised iron supplementation strategies that ensure both maternal and fetal well-being while reducing the burden of anemia-related complications.

## Materials and methods

### Study design

This was a randomised, comparative, parallel arm, active-controlled, open-label multicentric clinical study to evaluate the safety and efficacy of SunActive™ Fe versus Ferrous Ascorbate in pregnant women with IDA. Group A (test, *n* = 55) received SunActive™ Fe (27 mg), and Group B (control, *n* = 56) received Ferrous Ascorbate (100 mg) once daily for 4 weeks.

The study was conducted at three sites - Kala Hospital and Clinical Laboratory, Yeswanthpur, Bangalore, Karnataka, Al-Ameen Medical College Department of Obstetrics and Gynaecology, Vijayapur, Bijapur, Karnataka, and Fertility Associates Clinic, Khar West, Mumbai, Maharashtra. The study was initiated only after written approval was obtained from the Independent Ethics Committee that is Royal Pune Independent Ethics Committee, Pune (DCGI REF NO: ECR/45/Indt/MH/2013/RR-19) for all three sites. The clinical trial was registered with the Clinical Trial Registry-India (CTRI) under the registration number CTRI/2024/07/070773 [Registered on: 18/07/2024]. The study was conducted as per the approved protocol, Declaration of Helsinki, and Good Clinical Practices guidelines. Clinical trial data were collected between July 2024 and January 2025. Throughout the investigation, the authorised clinical procedure did not alter. The formulation of SunActive™ Fe tablets consists of EMFP, providing 27 mg of elemental iron, along with ascorbic acid 50 mg, vitamin B12 0.75 mcg, folic acid 250 mcg, and glycine 10 mg. Ferrous Ascorbate tablets contain Ferrous Ascorbate IP equivalent to 100 mg of elemental iron, complemented by folic acid IP 1.5 mg.

### Informed consent

Written informed consent was obtained from all participants before enrollment. Participants were informed about the objectives, methods, possible risks and benefits of the study and confidentiality policies, and the institutional ethics committee’s and study investigator’s contact details. To maintain participant confidentiality, all personal data and study information were securely archived. Anonymity of the participants was preserved in all publications and study reports. The data were only accessible to approved study personnel.

### Inclusion criteria

Pregnant females aged 20–35 years with a singleton intrauterine pregnancy (13–16 weeks), primi or multigravida, no comorbidities, Hb 9–10.5 g/dL, serum ferritin < 15 mcg/L, with or without anemia-related fatigue, able to provide consent and attend follow-ups, were eligible.

### Exclusion criteria

Pregnant women with gestational age < 13 or > 16 weeks, a history of complicated pregnancy or ongoing treatment for pregnancy complications (e.g., bleeding piles, excessive emesis, active peptic ulcer, diabetes, hypertension, eclampsia, thyroid disorders, multiple pregnancy), detectable fetal anomalies, or those on concomitant IDA therapy were ineligible. Participants unwilling to provide consent, follow up, or with any condition deemed unsuitable by the investigator were also excluded.

### Sample size

A total sample size of 102 completed cases is required to assess the study objective of achieving a non-inferiority between mean Hb change of test and control by at least 5% with 95% power and a 5% significance level, necessitating a minimum of 46 participants in each group with a 1:1 allocation ratio between test and comparator.

### Methodology

A total of 111 participants were randomised in a 1:1 ratio, and 108 completed the 4-week study. Participants were randomised using a computer-generated randomisation list developed by a qualified biostatistician. This list was generated prior to study initiation to ensure unbiased allocation.

On baseline visit (day one), patients received either one tablet of 27 mg SunActive™ Fe orally once daily before lunch or one tablet of 100 mg Ferrous Ascorbate orally once daily post lunch.

Drug adherence was assessed throughout the study, and if missed dosing for > 3 consecutive days or the total missed dose was > 6 during the 30-day period, the patient was considered a dropout. Patients were advised to continue the diet and exercise regimen as suggested by the investigator and were assessed for any adverse events during the study period.

Efficacy assessments were conducted at screening, baseline, and week 4, and included measurements of Hb, reticulocyte hemoglobin (RET-He) (a valuable diagnostic marker for assessing functional iron deficiency and early iron-restricted erythropoiesis. It provides a real-time evaluation of iron availability for RBC production, often detecting iron deficiency earlier than traditional markers like serum ferritin or Hb), ferritin, complete blood count (CBC), Fatigue severity was assessed using the Functional Assessment of Chronic Illness Therapy–Fatigue (FACIT-Fatigue) scale, where higher scores indicate lower fatigue severity, iron deficiency, and GI symptoms. Safety was evaluated through the monitoring of adverse events (AEs), serious adverse events (SAEs) and an overall tolerability & compliance assessment. Adverse events were recorded through participant self-reports and investigator observations during study visits. They were classified based on severity (mild, moderate, severe), seriousness, and relationship to the study intervention.

**Fig. 1:**
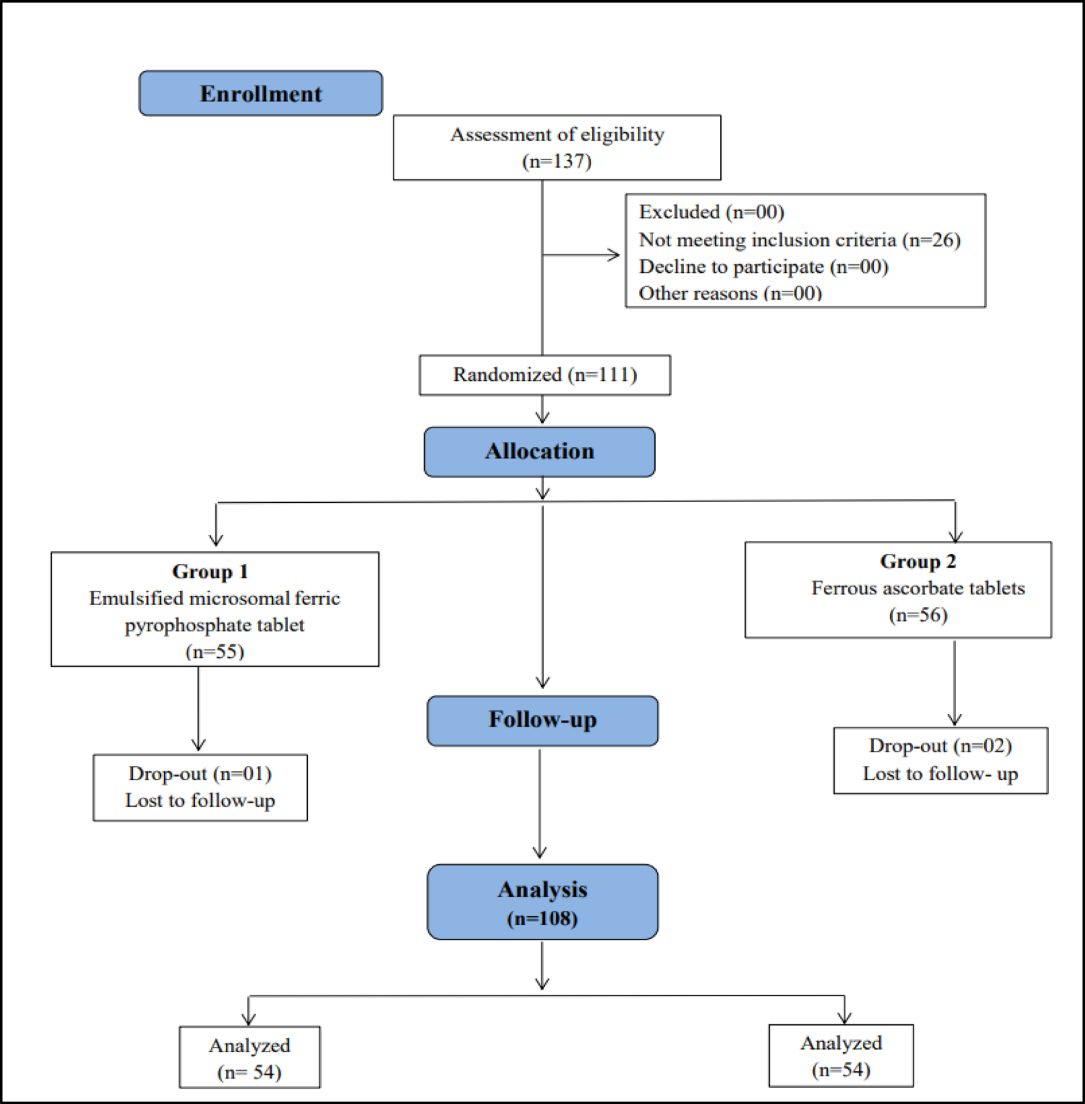
CONSORT flow diagram.

The CONSORT diagram is presented in Fig. 1.

### Statistical analysis

Age was summarised using mean ± SD with 95% CI and analysed via Student’s t-test. Normality was assessed by the Kolmogorov-Smirnov test. Changes in Hb, serum ferritin, serum iron, and RET-He were analysed using Student’s t-test, while fatigue severity and symptom changes were assessed using the Wilcoxon signed-rank test (within-group) and Mann-Whitney U test (between-group). Hematological parameters, compliance, and vitals were analysed via Student’s t-test, and adverse events were assessed using Fisher’s Exact Test. All analyses were performed using SPSS 10.0. The p-values ≤ 0.05 were considered to indicate statistical significance.

## Results

### Demographic characteristics

In this study, the age of the participants ranged from 20.00 to 35.00 years, with an average age being 25.61 years among the test group, which was comparable to 25.48 years in the control group, and the difference was not statistically significant. Data are summarised in Table 1.

**Table 1:** Demographic Details.

Parameters	Test group	Control group	*P* value
Number of cases	54	54	-
Age (years)	25.61 ± 03.78	25.48 ± 3.82	0.859

Data is represented as Mean ± S.D. The age data was analysed using the Student T-test.

### Assessment of changes in hemoglobin levels between groups

**Fig. 2:**
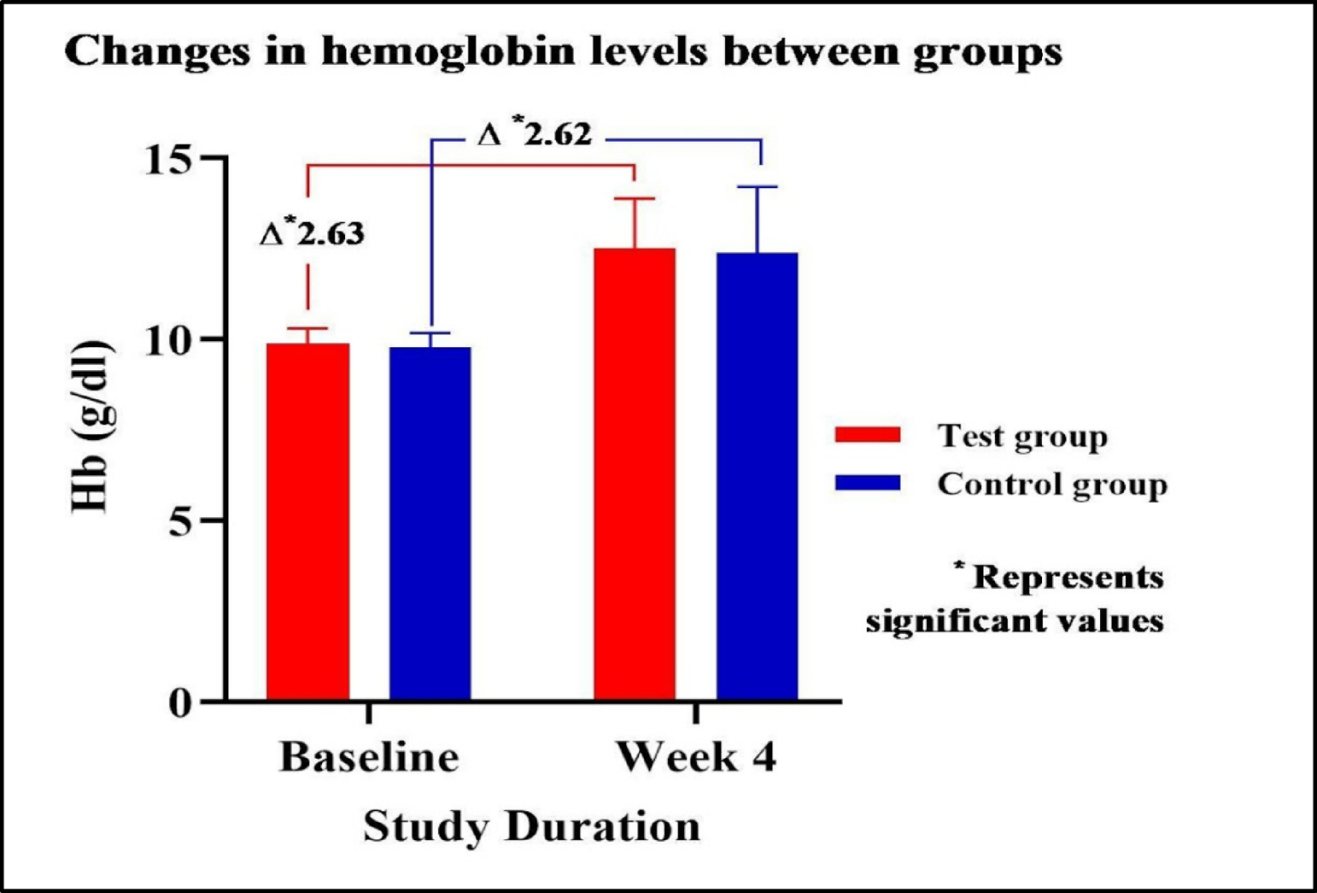
Changes in hemoglobin levels between groups.

**Fig. 3:**
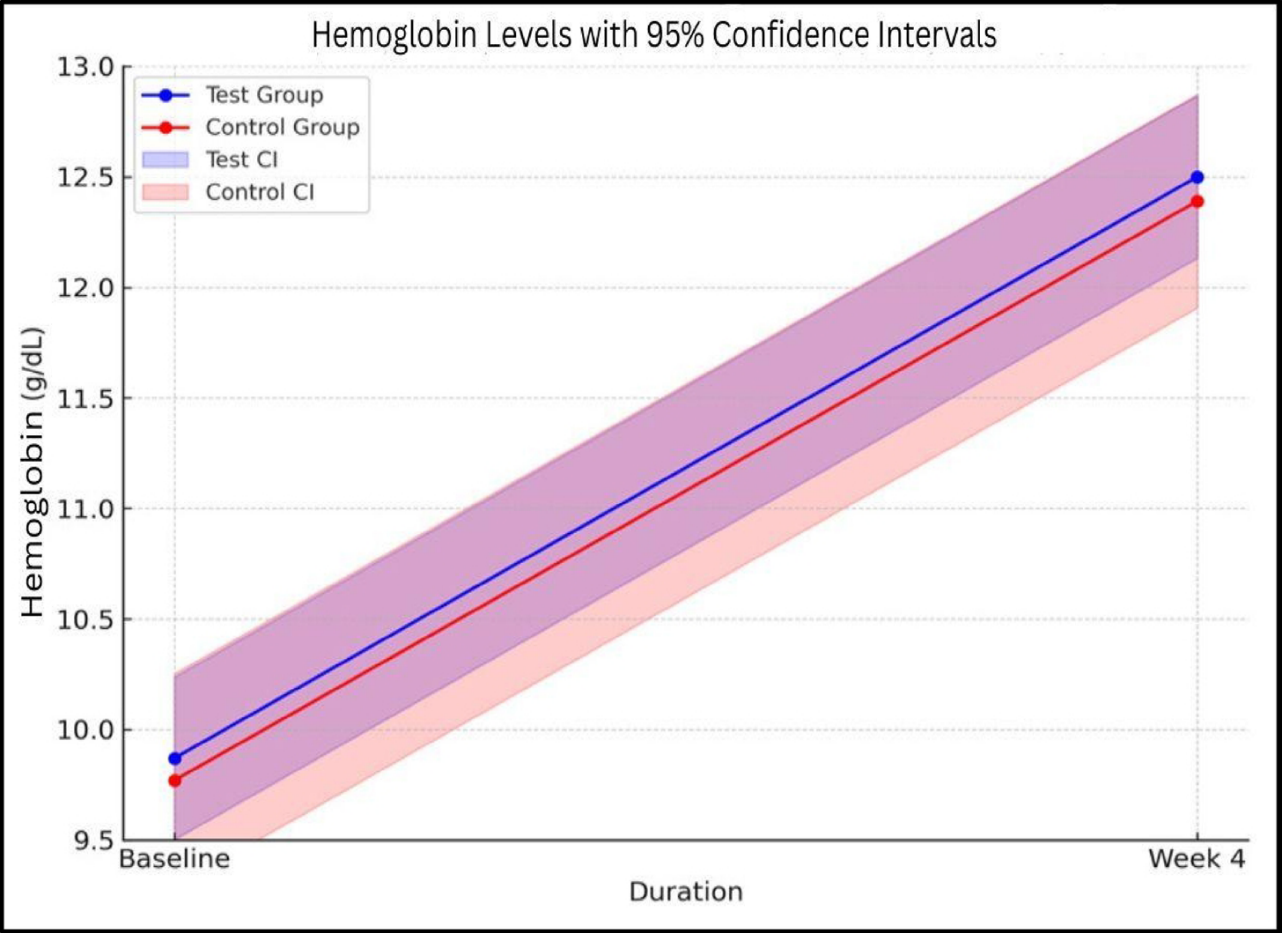
Depiction of confidence intervals of both groups visually supports the non-inferiority conclusion.

Both groups showed significant Hb improvements by week 4, with a Δ 2.63 increase in the test group and a Δ 2.62 in the control group, indicating comparable efficacy. The between-group difference was statistically not significant, suggesting comparative effectiveness. Data summarised in Table 2**&** Figs. 2 and 3. Since the mean values, confidence intervals (CI), and p-value indicate no significant difference between the test and control groups, the test treatment can be considered comparable to the control, at the predefined non-inferiority margin of 5% difference. The difference of 5% of the control group mean (12.39 g/dL) is − 0.6195 g/dL. The lower bound of the test group CI is -0.34 g/dL, which is more than the 5% difference from the control. The test treatment remains comparable to the control treatment under a 5% margin.

**Table 2:** Assessment of changes in hemoglobin levels between groups.

Duration	Test group	Control group	*P* value
Hemoglobin (g/dL)			
Baseline	09.87 ± 0.43	09.77 ± 0.40	0.213
Week 4	12.50 ± 1.38	12.39 ± 1.81	-
95% CI (Baseline)	[12.132–12.868]	[11.907–12.873]	-
95% CI (Week 4)	[12.132–12.868]	[11.907–12.873]	-
Mean diff (p value)	2.63 ± 1.25 (0.001)	2.62 ± 1.81 (0.001)	0.973 (NS)
Serum Ferritin (ng/mL)			
Baseline	12.04 ± 02.46	12.00 ± 02.51	0.933 (NS)
Week 4	61.09 ± 66.43	61.92 ± 61.51	-
95% CI (Baseline)	[11.384–12.696]	[11.331 − 12.669]	-
95% CI (Week 4)	[43.372–78.808]	[45.514 − 78.326]	-
Mean diff (p value)	49.05 ± 65.97 (0.001)	49.92 ± 60.54 (0.001)	0.943 (NS)
Reticular hemoglobin (pg/cell)			
Baseline	25.98 ± 1.25	26.22 ± 1.15	0.301 (NS)
Week 4	31.30 ± 3.10	30.47 ± 3.09	-
95% CI (Baseline)	[25.647–26.313]	[25.913–26.527]	-
95% CI (Week 4)	[30.473–32.127]	[29.646–31.294]	-
Mean diff (p value)	5.32 ± 3.50 (0.001)	4.25 ± 3.06 (0.001)	0.093 (NS)

Data is represented as Mean ± S.D. and 95% Confidence Interval. The data was analysed using the Student T-test.

### Assessment of changes in serum ferritin & reticular hemoglobin levels between groups

**Fig. 4:**
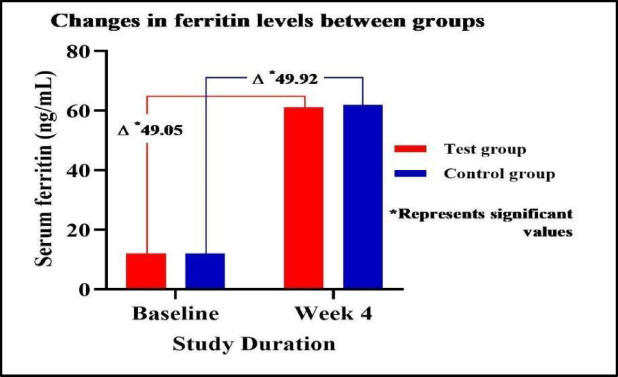
Changes in serum ferritin levels between groups.

At baseline, serum ferritin and RET-He levels were comparable between the test and control groups, with no significant difference. By week 4, both groups showed a significant increase in serum ferritin and RET-He levels from baseline. The between-group difference was not statistically significant, suggesting that both the test and control groups exhibited comparable improvements in serum ferritin and RET-He levels. Data summarised in Table 2**&** Fig. 4.

### Assessment of changes in fatigue severity score

**Fig. 5:**
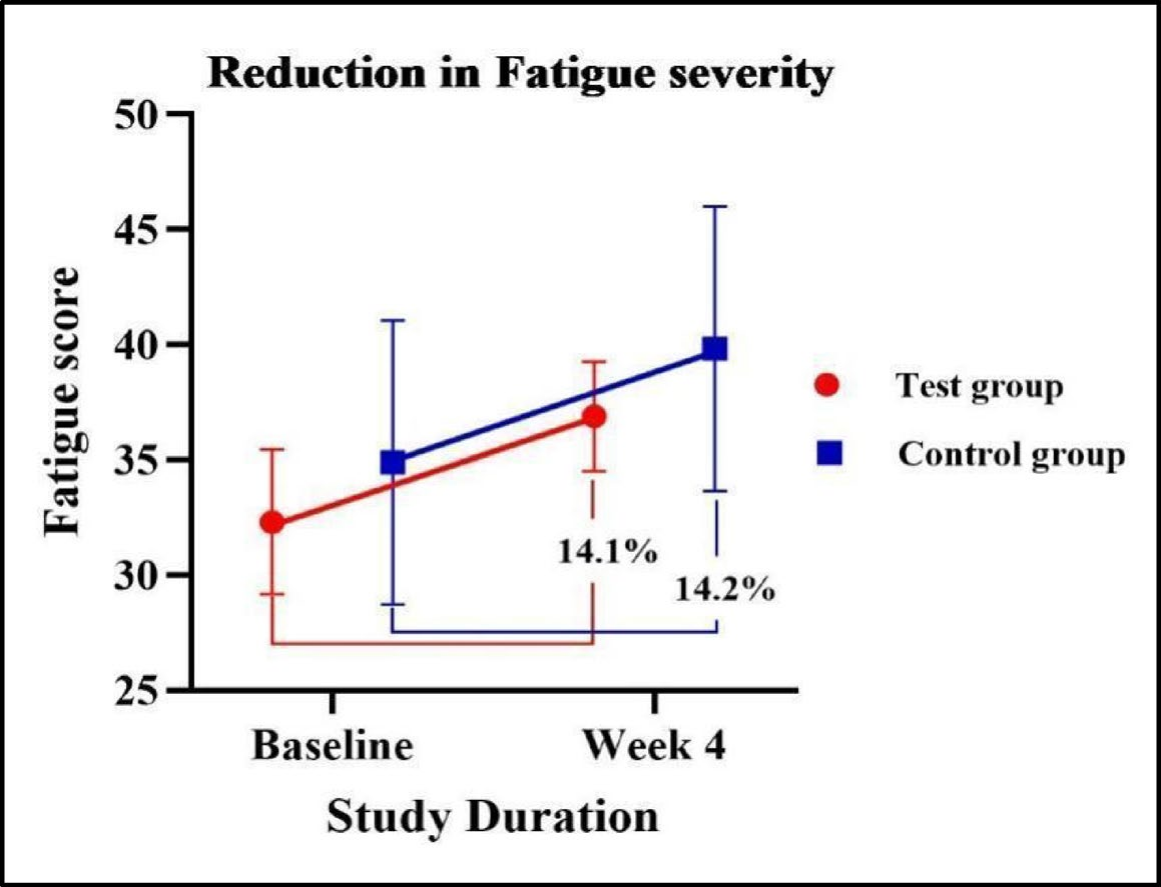
Reduction in fatigue severity.

After Week 4, both groups showed a comparable increase of 14.1% in the test group and 14.2% in the control group, with no significant difference between them. Data are summarised in Fig. 5.

### Assessment of changes in symptoms between the groups

At baseline, no significant differences were observed between groups. By Week 4, palpitations decreased by 16.13% in the test group (no change in control), and dizziness improved more in the test group (22.8%) vs. control (4.3%). Constipation and nausea were reported more in the control group.

### Assessment of adverse events

During the study, adverse events including nausea, dark stools, and hyperacidity were reported in 6 participants (11.1%) from the control group, while no such events occurred in the test group. Individual adverse events did not show any significant differences between groups. However, when total adverse events were compared, the difference was statistically significant, indicating better gastrointestinal tolerability of the test intervention compared to the control. All events were mild, transient, and resolved spontaneously without requiring rescue medication or discontinuation of treatment. Data are summarised in Table 3. The mean compliance of treatment at Week 4 was comparable between the Test (99.88%) and Control (99.75%) groups, with no significant differences.

**Table 3:** Assessment of adverse Events.

Adverse Events	Test group (*N* = 54)		Control group (*N* = 54)		*P*-value
	No.	%	No.	%	
Hyperacidity	-	-	2	3.7	-
Nausea	-	-	3	5.5	-
Dark stools	-	-	1	1.9	-
Total No. of Events	-	-	6	-	-
Total No. of Patients	-	-	6	11.1	*0.028

Data are presented as the number of participants (n) and the percentage (%). Fisher’s Exact Test was used for analysis. * represents a significant P-value at *p* < 0.05.

### Assessment of comparison of mean hematological parameters & vital signs between the groups

At baseline, the mean total RBC count was similar in both groups (3.86 mil/cu.mm in Test, 3.87 mil/cu.mm in Control), with comparable increases by Week 4 (9.1% in Test, 9.8% in Control). All the other hematological parameters were within normal clinical ranges. Vital parameters like blood pressure, heart rate, body temperature, and respiratory rate remained stable and within normal physiological ranges for both groups.

## Discussion

This open-label, randomised, comparative study was conducted to evaluate the efficacy and safety of SunActive™ Fe versus Ferrous Ascorbate tablets in pregnant women diagnosed with IDA. To the best of our knowledge, this trial is the first of its kind to conduct a direct comparative analysis between SunActive™ Fe, a unique form of microsomal iron, and Ferrous Ascorbate, a commonly used iron supplement, within the specific demographic of pregnant women suffering from IDA.

Eligible participants were randomised in a 1:1 ratio to one of the following treatment groups: the test group received an iron supplement SunActive™ Fe tablet for 4 weeks (27 mg), while a control group received Ferrous Ascorbate tablets for 4 weeks (100 mg).

The efficacy and safety profile of SunActive™ Fe was recently evaluated in managing iron deficiency anemia in pregnant women^13^. Patients showed high treatment compliance and no adverse events related to the investigational product. Our current results are consistent with previous findings, reinforcing the effectiveness of this iron formulation.

Previously published studies have demonstrated that oral supplementation with SunActive™ Fe is highly effective, with no reported side effects and exceptional patient compliance^18,19^.

*Gogineni et al.* (2015) conducted a comparative study evaluating oral Ferrous Ascorbate (100 mg) and intravenous iron sucrose for prophylactic iron therapy in pregnancy. While both groups showed similar improvements in Hb levels, the study highlighted a significantly higher incidence of adverse drug reactions with Ferrous Ascorbate, with 66% of participants experiencing gastric side effects compared to only 4% in the iron sucrose group^20^. These findings align with our study, where Ferrous Ascorbate also showed higher gastric adverse events.

A recently published study by *Kulkarni et al.* (2024) in healthy, non-pregnant females demonstrated that SunActive™ Fe, effectively increases iron and Hb levels while being well tolerated. Notably, SunActive™ Fe at just one-third the concentration of traditional iron supplementation (100 mg) exhibited comparable efficacy in boosting iron levels by day 16. Hb levels showed a greater increase as early as day 8 in the SunActive™ Fe group compared to those receiving Ferrous Ascorbate, highlighting its rapid and efficient absorption^21^. These results are consistent with our research, demonstrating that SunActive™ Fe, at merely one-third of the dosage, provides efficacy similar to that of 100 mg.

Although the difference in RET-He between groups was not statistically significant, the numerically greater increase observed in the test group, despite the substantially lower elemental iron dose may indicate efficient iron utilization. RET-He is a sensitive marker of functional iron availability and often responds earlier than hemoglobin^22^. In pregnant women, its diagnostic performance has been validated and shown to outperform traditional markers^23^. Findings similar to those in our study were reported by *Schoorl et al.* (2012), who observed significant increases in reticulocyte counts, RET-He, and hemoglobin after four weeks of ferrous fumarate (~ 200 mg elemental iron/day) in pregnant women with suspected iron-deficient erythropoiesis^24^. These results suggest that EMFP may support efficient iron utilization at lower doses, which could be particularly advantageous during the second trimester of pregnancy.

This study’s strengths include its prospective design with parallel arms, enabling a direct comparison between the test and control groups, and marking the first such study in pregnant women with IDA. This comparison provides valuable insights into the efficacy, safety, and tolerability of both formulations, where SunActive™ Fe at just one-third the dose of Ferrous Ascorbate achieves comparable efficacy in improving Hb, RBCs, and serum ferritin levels while significantly reducing fatigue. SunActive™ Fe presents a promising therapeutic strategy for pregnant women, balancing efficacy with reduced side effects, which is crucial for improving maternal health outcomes. Nevertheless, limitations such as the restricted sample size, brief duration, and open-label design may influence the generalizability of the findings. Although participants received guidance on general dietary recommendations, complete control over their diet was not maintained. The study was conducted on a pregnant population, and a placebo arm was omitted to ensure subjects received iron supplementation. C-Reactive Protein was not measured and it is possible that having that value could also lend additional insight. Additional micronutrients in the SunActive™ Fe formulation, specifically vitamin B12 and ascorbic acid may have exerted a synergistic effect on hematological outcomes. While this may confound a direct comparison focused solely on iron efficacy, it also mirrors real-world treatment strategies for anemia, which often involve combined nutrient support. An intravenous arm was not incorporated as participants did not exhibit severe anemia. Future research should include larger, blinded trials using formulations with matched micronutrient content to validate these findings and investigate the long-term efficacy, safety, and impact on comorbid conditions associated with iron supplements in pregnant women with IDA.

SunActive™ Fe achieved comparable efficacy at only one-third the elemental iron dose (27 mg vs. 100 mg) while demonstrating significantly better gastrointestinal tolerability and adverse event profile than ferrous ascorbate. These attributes are highly relevant in clinical practice, as tolerability is often the primary factor driving discontinuation of oral iron therapy.

Although the unit cost of encapsulated irons are higher than conventional irons, their better safety profile, lower dose requirement, and potential to improve compliance suggest they may provide a clinical advantage that could justify consideration despite the cost difference. Future pharmacoeconomic analyses are warranted. Improved adherence through reduced side effects could have a greater impact on therapeutic success than absolute cost alone.

## Conclusion

This study highlights the safety profile and superior tolerability of SunActive™ Fe. It was seen that at week 4, both SunActive™ Fe and Ferrous Ascorbate demonstrated similar efficacy in improving Hb, serum ferritin, serum iron, fatigue severity, and RET-He levels in pregnant women with iron deficiency anemia. However, adverse events, particularly gastrointestinal symptoms such as nausea, hyperacidity, & dark stools, were reported only in the Ferrous Ascorbate group.

## Data Availability

The datasets generated and/or analysed during the current study are not publicly available due to intellectual property constraints, but are available from the corresponding author on reasonable request.
